# Two New Compounds from *Schisandra propinqua* var. *propinqua*

**DOI:** 10.1007/s13659-017-0129-7

**Published:** 2017-05-03

**Authors:** Miao Liu, Zheng-Xi Hu, Yuan-Qing Luo, Min Zhou, Wei-Guang Wang, Xiao-Nian Li, Xue Du, Jian-Xin Pu, Han-Dong Sun

**Affiliations:** 1 State Key Laboratory of Phytochemistry and Plant Resources in West China, Kunming Institute of Botany, Chinese Academy of Sciences, Kunming, 650201 People’s Republic of China; 2 University of Chinese Academy of Sciences, Beijing, 100049 People’s Republic of China; 3 Hubei Key Laboratory of Natural Medicinal Chemistry and Resource Evaluation, School of Pharmacy, Tongji Medical College, Huazhong University of Science and Technology, Wuhan, 430030 People’s Republic of China

**Keywords:** *Schisandra propinqua* var. *propinqua*, Bergamotane-type sesquiterpenoid, Lignan, Absolute configuration, Cytotoxicity

## Abstract

**Abstract:**

Schisanpropinoic acid (**1**), a new bergamotane sesquiterpenoid, and schisanpropinin (**2**), a new tetrahydrofuran lignan with a rare epoxyethane unit, were identified from the stems and leaves of *Schisandra propinqua* var. *propinqua*. Their structures were determined based on comprehensive spectroscopic and mass spectrometric analysis. The absolute configuration of **1** was determined by X-ray analysis. Compounds **1** and **2** were tested for their cytotoxic activity against five human tumor cell lines.

**Graphical Abstract:**

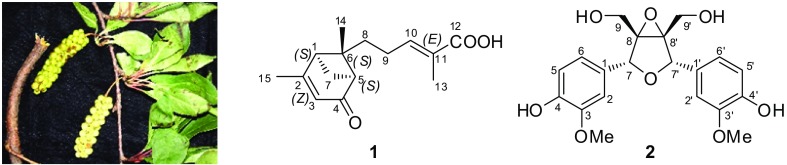

**Electronic supplementary material:**

The online version of this article (doi:10.1007/s13659-017-0129-7) contains supplementary material, which is available to authorized users.

## Introduction

Schisandraceae family are a group of economically and medicinally valuable climbing woody vine plants, some of which are used as traditional Chinese medicines in Chinese folk for over 2000 years [1]. Since 1970s, researches on the plants of Schisandraceae family have been a hot topic [2]. In 2003, micrandilactone A, a highly oxygenated, skeleton rearranged, polycyclic nortritepenoid, was reported from *S. micrantha* by our group [3]. This new finding pushed the studies on the plants of Shisandraceae to a new climax. As a result, last ten years have seen the isolation and identification of large numbers of *Schisandra* nortriterpenoids (SNTs) from the Schisandraceae family [2, 4].

**Fig. 1:**
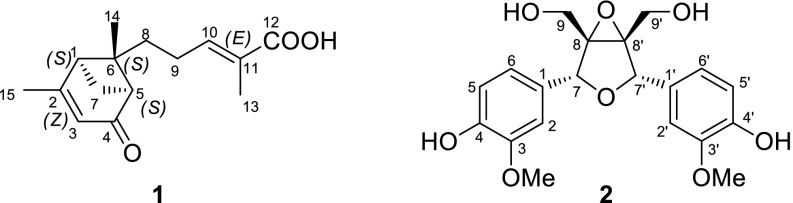
Structures of compounds **1** and **2**

*S. propinqua* (Wall.) Baill. var. *propinqua* is indigenous in Yunnan Province. Previous chemical investigation into this species has led to the identification of a series of new terpenoids and lignans, such as propindilactones E-O [5, 6], lanopropic acid [7], methylisogomisin O [8], and propindilactones T and U [9]. Our further study on this species, which was collected from Weixi county, Yunnan Province, People’s Republic of China to discover new compounds with interesting bioactivity led to the discovery of a new sesquiterpenoid and a new lignan (Fig. 1). This article discribes the isolation, structure elucidation and bioactivities of these two new compounds.

## Results and Discussion

**Fig. 2:**
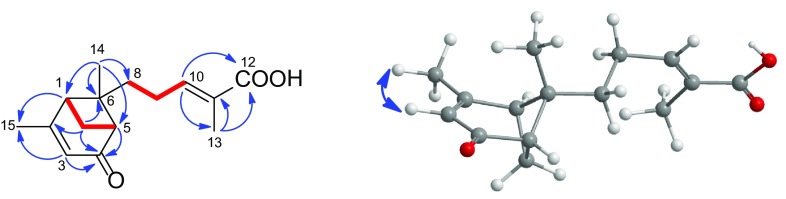
Key HMBC (*blue arrows* H→C), ^1^H–^1^H COSY (*red lines*), and ROESY correlations (*blue double-headed arrow*) of **1**

Compound **1**, obtained as colorless crystals (MeOH). Its IR absorptions at 1701 and 1642 cm^−1^ implied the existence of C=O and C=C groups. The molecular formula was determined as C_15_H_20_O_3_ from the HRESIMS ion at *m/z* 271.1304 [M + Na]^+^ (calcd for C_15_H_20_O_3_Na 271.1305) with 6 degrees of unsaturation. The ^13^C NMR spectrum of **1** displayed only 13 carbon signals. A careful analysis of 1D NMR data (Table 1) and HRESIMS spectrum combined with the HSQC and HMBC spectra (Fig. 2) allowed the assignment of **1** as a sesquiterpenoid with three singlet methyls, three methylenes, two methines, one quaternary carbon, two trisubstituted double bond, one ester carboxyl groups and one carbonyl group. Thus, **1** was a sesquiterpenoid with two rings. The ^1^H–^1^H COSY correlations between H-8/H-9/H-10 (Fig. 2) and the HMBC correlations from H-10 (*δ*_H_ 7.17, 1H, t, *J* = 7.0 Hz) to C-13 (*δ*_C_ 13.1) and C-12 (*δ*_C_ 171.0), and from H-9 (*δ*_H_ 1.87, 2H, m) to C-6 (*δ*_C_ 56.6) proved the presence of the side chain. Furthermore, the HMBC correlations from H-1 (*δ*_H_ 2.34, 1H, br t, *J* = 6.0 Hz) and H-5 (*δ*_H_ 2.83, 1H, br t, *J* = 6.0 Hz) to C-3 (*δ*_C_ 122.0) and C-14 (*δ*_C_ 19.1), from H-1 and H-3 (*δ*_H_ 5.87, 1H, m) to C-15 (*δ*_C_ 23.4), from H-3 and H-5 to C-4 (*δ*_C_ 202.5), and from H-9 to C-6 revealing the six-membered ring unit. The ^1^H-^1^H COSY correlations of H-1/H-5, H-5/H-7, and H-1/H-7 (Fig. 2) seemed to disclose the existence of cyclopropane unit, while it was confronted with the HMBC correlations from H-7 (*δ*_H_ 1.92, 1H, d, *J* = 9.3 Hz; 2.63, 1H, dt, *J* = 9.3, 6.0 Hz) to C-2 (*δ*_C_ 170.0), C-4, and C-6. Given a special long-range spin–spin coupling of H-1/H-5 (^4^ *J*_1,5_ = 6.0 Hz) that subsequently occurred in the cyclobutane unit [10, 11], a cyclobutane unit other than cyclopropane might be existed. This deduction was consistent with all the above-mentioned HMBC correlations. Herein, a structure of cyclobutane adjoined with cyclohexane, along with a side chain unit was elucidated. Compound **1** was a bergamotane-type sesquiterpenoid [12, 13].

**Table 1:** NMR data of **1** and **2** (C_5_D_5_N, *δ* in ppm, *J* in Hz)

No.	**1** ^a^		No.	**2** ^b^	
	*δ*_H_	*δ*_C_		*δ*_H_	*δ*_C_
1	2.34, 1H (br t, 6.0)	48.7, d	1,1′		129.9, s
2		170.0, s	2,2′	7.54, 1H (d, 1.8)	113.2, d
3	5.87, 1H, m	122.0, d	3,3′		148.6, s
4		202.5, s	4,4′		148.2, s
5	2.83, 1H (br t, 6.0)	56.4, d	5,5′	7.30, 1H (d, 8.0)	116.2, d
6		56.6, s	6,6′	7.34, 1H (dd, 8.0, 1.8)	121.6, d
7	1.92, 1H (d, 9.3) 2.63, 1H (dt, 9.3, 6.0)	40.8, d	7,7′	5.47, 1H (s)	88.9 d
8	2.16, 2H (m)	37.7, t	8,8′		89.4 s
9	1.87, 2H (m)	24.5, t	9,9′	4.54, 2H (s)	77.0 t
10	7.17, 1H (t, 7.0)	141.4, d	OMe	3.72, 3H (s)	56.1, q
11		129.9, s			
12		171.0, s			
13	2.08, 3H (s)	13.1, q			
14	1.00, 3H (s)	19.1, q			
15	1.85, 3H (d, 1.5)	23.4, q			

The assignments were based on HSQC, ^1^H–^1^H COSY, and HMBC experiments

^a^ ^1^H and ^13^C NMR data were recorded at 500 and 125 MHz, respectively

^b^ ^1^H and ^13^C NMR data were recorded at 600 and 150 MHz, respectively

**Fig. 3:**
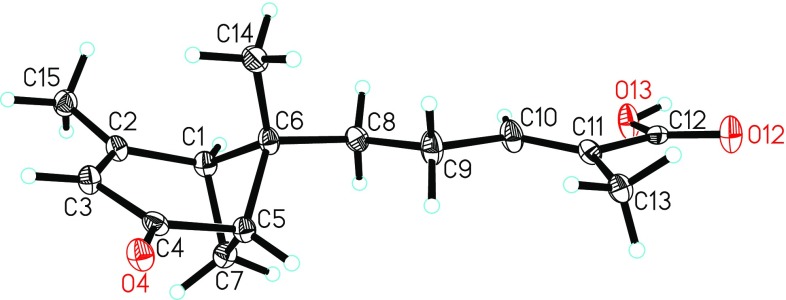
X-ray structure of **1**

Even though the structure of **1** was deduced from extensive spectroscopic analysis, no available correlations in the ROESY spectrum were observed (Fig. 2). Thus, the structure of **1**, especially the absolute configuration still needed to be further confirmed. Luckily, a single-crystal X-ray diffraction was performed successfully (Fig. 3). Through structural refinement, the Flack parameter with 0.0(4) and the Hooft parameter at 0.03(17) for 1371 Bijvoet pairs [14, 15], allowed an explicit assignment of the absolute configuration of **1** as 1*S*, 5*S*, 6*S*. Finally, the structure of **1** was determined, and named schisanpropinoic acid.

It is worth noting that **1** was the first sesquiterpenoid with a bergamotane skeleton from Schisandraceae family. In addition, bergamotane sesquiterpenoids were infrequently reported in plants [12, 13, 16–18], and recent discoveries of new bergamotane-type sesquiterpenoids were resulted from fungi resources [19–26].

**Fig. 4:**
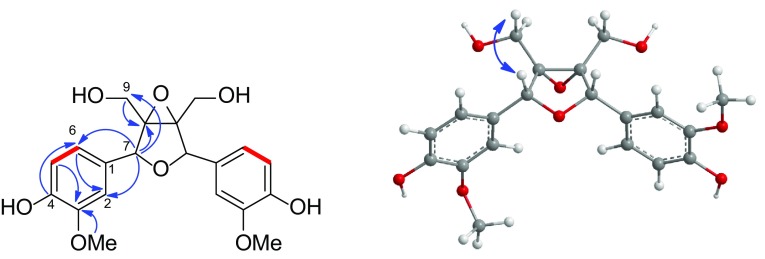
Key HMBC (*blue arrows* H→C), ^1^H-^1^H COSY (*red lines*), and ROESY correlations (*blue double-headed arrow*) of **2**

Compound **2**, obtained as a yellowish solid (MeOH). Its IR absorptions at 3424, 1607, 1518 and 1462 cm^−1^ implied the presence of OH and phenyl groups. Its molecular formula was determined as C_20_H_22_O_8_ by HRESIMS at *m/z* 389.1237 [M − H]^−^ (calcd for C_20_H_21_O_8_ 389.1242), indicating 10 indices of hydrogen deficiency of **2**. ^13^C NMR spectrum (Table 1) displayed only ten carbon signals (six aromatic carbons, one oxygenated *sp* ^3^ quaternary carbon, one oxygenated *sp* ^3^ methine, one oxygenated methylene, and one methoxy group), suggesting the symmetrical structure of **2**. The ^1^H NMR signals (Table 1) at *δ*_H_ 7.34 (1H, dd, *J* = 8.0, 1.8 Hz), 7.54 (1H, d, *J* = 1.8 Hz), and 7.30 (1H, d, *J* = 8.0 Hz) indicating the 1,2,4-trisubstituted benzene. This deduction was also confirmed by the ^1^H–^1^H COSY (Fig. 4) correlation between H-5 and H-6, as well as the HMBC correlations (Fig. 4) from H-7 (*δ*_H_ 5.47, 1H, s) to C-1 (*δ*_C_ 129.9), C-2 (*δ*_C_ 113.2) and C-6 (*δ*_C_ 121.6). Taking the lignans from Schisandraceae family into consideration, the above-mentioned data indicated that **2** is identical to the characteristics of a 4*H*-furan-type lignan with an extra oxygen-containing ring. The HMBC correlations from H-2 and H-6 to C-4, and from methoxyl (*δ*_H_ 3.72, 3H, s) to C-3 (*δ*_C_ 148.6) suggested that the methoxy group was located at C-3 rather than C-4. The oxygen-containing ring was deduced to be formed between C-8 and C-8′ by HMBC correlations from H-7 to C-8 (*δ*_C_ 89.4), from H-9 (*δ*_H_ 4.54 2H, s) to C-8, and from H-7 to C-9 (*δ*_C_ 77.0). Thus, the planar structure of **2** was determined. In the ROESY spectrum, both the correlation (Fig. 4) between H-9 and H-7 as well as the confusing correlations of H-2, H-6 and H-9 were observed. Then, a computer-aided 3D molecular model study of two possible relative structure of **2** was conducted. As a result, when H-9 and H-7 were at a same orientation, the internuclear distance between H-9 and H-7 was less than 3.0 Å. On the contrary, the internuclear distance was lager than 3.0 Å. At the same time, the confusing ROESY correlations of H-2, H-6 with H-9 could be explained by the free rotation of the C_1_–C_7_ bond. Considering all these factors, the chemical structure of **2** with a relative configuration was determined (Fig. 1). **2** was named schisanpropinin and was a tetrahydrofuran lignan with a rare epoxyethane unit from Schisandraceae family [27].

Compounds **1** and **2** were tested for their cytotoxicity against HL-60, A-549, SMMC-7721, MCF-7, and SW-480 human tumor cell lines by MTS methods using cisplatin and paclitaxel as positive controls. Neither of them showed cytotoxic any activity against all cell lines with IC_50_ values >40 µM (cisplatin: IC_50_ = 3.13, 19.05, 13.07, 23.64, and 11.25 µM, respectively, paclitaxel: all <0.008 µM).

## Experimental

### General

1D and 2D NMR spectra were recorded on Bruker DRX 500 or 600 spectrometers using C_5_D_5_N as the internal standard. Chemical shifts (*δ*) are expressed in ppm relative to the C_5_D_5_N signals. HRESIMS was performed on an API QSTAR Pulsar i spectrometer. UV spectra were obtained on a Shimadzu UV-2401PC spectrophotometer. IR spectra were obtained on a Bruker Tensor-27 FT-IR spectrometer using KBr pellets. Optical rotations were measured in MeOH with JASCO P-1020 polarimeters. Column chromatography (CC) was performed with silica gel (100–200 mesh; Qingdao Marine Chemical, Inc., Qingdao, People’s Republic of China), MCI gel (CHP20P, 75–150 μm, Mitsubishi Chemical Corporation, Tokyo, Japan). Semi-preparative HPLC was performed on an Agilent 1200 liquid chromatograph with a Zorbax SB-C18 (9.4 mm × 250 mm) column. Fractions were monitored by thin layer chromatography, spots were visualized by UV light (254 and 365 nm) and by heating silica gel plates sprayed with 10% H_2_SO_4_ in EtOH. All solvents used in column chromatography were distilled including petroleum ether (60–90 °C).

### Plant Material

The stems and leaves of *S. propinqua* var. *propinqua* were collected in Weixi county, Yunnan Province, People’s Republic of China, in July 2013 and identified by Prof. Xi-Wen Li at Kunming Institute of Botany. A voucher specimen (KIB 2013071801) has been deposited in the State Key Laboratory of Phytochemistry and Plant Resources in West China, Kunming Institute of Botany, Chinese Academy of Sciences.

### Extraction and Isolation

The air-dried and powdered stems and leaves of *S. propinqua* var. *propinqua* (21.0 kg) were extracted with 70% aqueous acetone (4 × 40 L, 3 days each) at room temperature. The extract was distilled under reduced pressure to remove acetone, and then partitioned between EtOAc and H_2_O to afford the EtOAc fraction. EtOAc fraction (690 g) was applied to a silica gel column and eluted with CHCl_3_/Me_2_CO (1:0, 9:1 8:2, 7:3, 1:1, 0:1) to give 6 fractions A-F.

Fraction B (9:1, 153 g) was decolorized on MCI gel with 90% MeOH/H_2_O and a repeated MCI gel CC with MeOH/H_2_O (60:40, 80:20, 100:0) to obtain B_1_–B_3_. Fraction B_2_ (1.6 g) was separated on RP-18 with MeOH/H_2_O (40:60, 45:55, 50:50, 55:45, 60:40, 65:35, 70:30, 100:0) to obtain B_21_–B_28_. B_21_ (58 mg) was purified by semi-preparative HPLC to afford **1** (16 mg).

Fraction D (7:3, 99 g) was decolorized on MCI gel with 90% MeOH/H_2_O to remove pigments and then on RP-18 with MeOH/H_2_O (30:70, 40:60, 50:50, 60:40, 70:30, 100:0) to afford fractions D_1_–D_6_. D_2_ (8.5 g) was separated by repeated RP-18 with MeOH/H_2_O (30:70, 35:65, 40:60, 45:55, 50:50, 60:40, 100:0) to afford D_21_–D_25_, and D_22_ (1.1 g) was separated by silica gel CC using light petroleum/acetone and separated out **2** (10 mg).

### The Cytotoxicity Assay

The human tumor cell lines HL-60, SMMC-7721, A-549, MCF-7, and SW-480 were used in the cytotoxic assay. These cell lines were obtained from ATCC (Manassas, VA, USA). Cells were cultured in RMPI-1640 or DMEM medium (Biological Industries, Kibbutz Beit-Haemek, Israel) supplemented with 10% fetal bovine serum (Biological Industries) at 37 °C in a humidified atmosphere with 5% CO_2_. The cytotoxicity assay was evaluated by the 3-(4,5-dimethylthiazol-2-yl)-5-(3-carboxymethoxyphenyl)-2-(4-sulfophenyl)-2H-tetrazolium, inner salt (MTS) (Promega, Madison, WI, USA) assay [28]. Briefly, cells were seeded into each well of a 96-well cell culture plate. After 12 h of incubation at 37 °C, the test compound (40 μM) was added. After incubated for 48 h, cells were subjected to the MTS assay [29, 30]. Compounds with a growth inhibition rate of 50% were further evaluated at concentrations of 0.064, 0.32, 1.6, 8, and 40 μM in triplicate, with cisplatin and paclitaxel (Sigma, St. Louis, MO, USA) as positive controls. The IC_50_ value of each compound was calculated with Reed and Muench’s method [31].

Schisanpropinoic acid (**1**): colorless crystals (MeOH). $\left[ \alpha \right]_{\text{D}}^{20}$ −167.0 (*c* 0.12, MeOH). UV (MeOH) *λ*_max_ (log *ε*) 250 (3.85), 209 (4.18) nm. IR (KBr) *v*_max_ 2983, 2932, 1701, 1642, 1432, 1381, 1342, 1239, 1207, 1129, 1306, 902 cm^−1^. ESIMS *m/z* 271 [M + Na]^+^, HRESIMS *m/z* 271.1304 [M + Na]^+^ (calcd for C_15_H_20_O_3_Na 271.1305). ^1^H NMR (C_5_D_5_N, 500 MHz) and ^13^C NMR (C_5_D_5_N, 125 MHz), see Table 1.

*Crystallographic data for* ***1***: C_15_H_20_O_3_, *M* = 248.31, monoclinic, *a* = 7.1516(7) Å, *b* = 23.527(2) Å, *c* = 7.8731(7) Å, *α* = 90.00°, *β* = 91.136(6)°, *γ* = 90.00°, *V* = 1324.5(2) Å^3^, *T* = 100(2) K, space group *P*21, *Z* = 4, *μ*(CuKα) = 0.687 mm^−1^, 6450 reflections measured, 3642 independent reflections (*R*_*int*_ = 0.0501). The final *R*_*1*_ values were 0.0653 (*I* > 2*σ*(*I*)). The final *wR*(*F* ^2^) values were 0.1771 (*I* > 2*σ*(*I*)). The final *R*_*1*_ values were 0.0661 (all data). The final *wR*(*F* ^2^) values were 0.1777 (all data). The goodness of fit on *F* ^2^ was 1.167. Flack parameter = 0.0 (4). The Hooft parameter is 0.03 (17) for 1371 Bijvoet pairs. Crystallographic data for the structure of **1** have been deposited in the Cambridge Crystallographic Data Centre (deposition number CCDC 1528224). Copies of the data can be obtained free of charge from the CCDC via www.ccdc.cam.ac.uk.

Schisanpropinin (**2**): yellowish solid, $\left[ \alpha \right]_{\text{D}}^{20}$ −31.2 (*c* 0.11, MeOH), UV (MeOH) *λ*_max_ (log *ε*) 286 (4.06), 205 (4.96) nm, IR (KBr) *v*_max_ 3424, 2928, 2856, 1607, 1518, 1463, 1431, 1383, 1277, 1160, 1071, 1031, 785 cm^−1^. ESIMS *m/z* 389 [M − H]^−^, HRESIMS *m/z* 389.1237 [M − H]^−^ (calcd for C_20_H_21_O_8_ 389.1242). ^1^H NMR (C_5_D_5_N, 600 MHz) and ^13^C NMR (C_5_D_5_N, 150 MHz), see Table 1.

## Electronic supplementary material

Below is the link to the electronic supplementary material.

(PDF 2,239 kb)
