# Supplementary material for: Two New Compounds from Schisandra propinqua var. propinqua
Source: Nat Prod Bioprospect. 2017 May 3;7(3):257–62. doi: 10.1007/s13659-017-0129-7 (PMC5481274; doi:10.1007/s13659-017-0129-7)

Supplementary data for

Two new compounds from *Schisandra propinqua* var. *propinqua*

Miao Liu[a,b], Zheng-Xi Hu[c], Yuan-Qing Luo[a,b], Min Zhou[a,b], Wei-Guang Wang[a], Xiao-Nian Li[a], Xue Du[a], Jian-Xin Pu*[a] and Han-Dong Sun[a]

[a] *State Key Laboratory of Phytochemistry and Plant Resources in West China, Kunming Institute of Botany, Chinese Academy of Sciences, Kunming 650201, People's Republic of China*

[b] *University of Chinese Academy of Sciences, Beijing 100049, People's Republic of China*

[c] *Hubei Key Laboratory of Natural Medicinal Chemistry and Resource Evaluation, School of Pharmacy, Tongji Medical College, Huazhong University of Science and Technology, Wuhan 430030, People's Republic of China Huazhong University*

Content

Fig. S1 HRESIMS spectrum of Schisanpropinoic acid (**1**)....2
Fig. S2 ^{1}H NMR spectrum (C_5D_5N, 500MHz) of Schisanpropinoic acid (**1**)....3
Fig. S3 ^{13}C NMR spectrum (C_5D_5N, 125MHz) of Schisanpropinoic acid (**1**)....4
Fig. S4 HSQC spectrum of Schisanpropinoic acid (**1**)....5
Fig. S5 ^{1}H-^{1}H COSY spectrum of Schisanpropinoic acid (**1**)....6
Fig. S6 HMBC spectrum of Schisanpropinoic acid (**1**)....7
Fig. S7 ROESY spectrum of Schisanpropinoic acid (**1**)....8
Fig. S8 UV spectrum of Schisanpropinoic acid (**1**)....9
Fig. S9 IR spectrum of Schisanpropinoic acid (**1**)....10
X-ray crystallographic data for **1**....11
Fig. S10 HRESIMS spectrum of schisanpropinin (**2**)....13
Fig. S11 ^{1}H NMR spectrum (C_5D_5N, 600MHz) of schisanpropinin (**2**)....14
Fig. S12 ^{13}C NMR spectrum (C_5D_5N, 150MHz) of schisanpropinin (**2**)....15
Fig. S13 HSQC spectrum of schisanpropinin (**2**)....16
Fig. S14 ^{1}H-^{1}H COSY spectrum of schisanpropinin (**2**)....17
Fig. S15 HMBC spectrum of schisanpropinin (**2**)....18
Fig. S16 ROESY spectrum of schisanpropinin (**2**)....19
Fig. S17 UV spectrum of schisanpropinin (**2**)....20
Fig. S18 IR spectrum of schisanpropinin (**2**)....21

Fig. S1 HRESIMS spectrum of Schisanpropinoic acid (**1**)

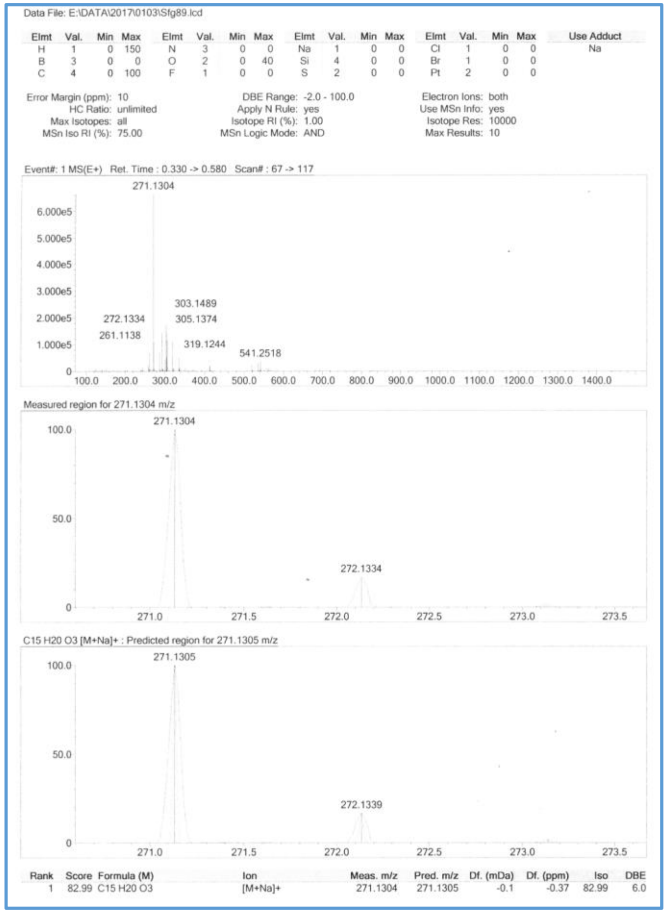

Fig. S2 ^{1}H NMR spectrum (C_5D_5N, 500MHz) of Schisanpropinoic acid (**1**)

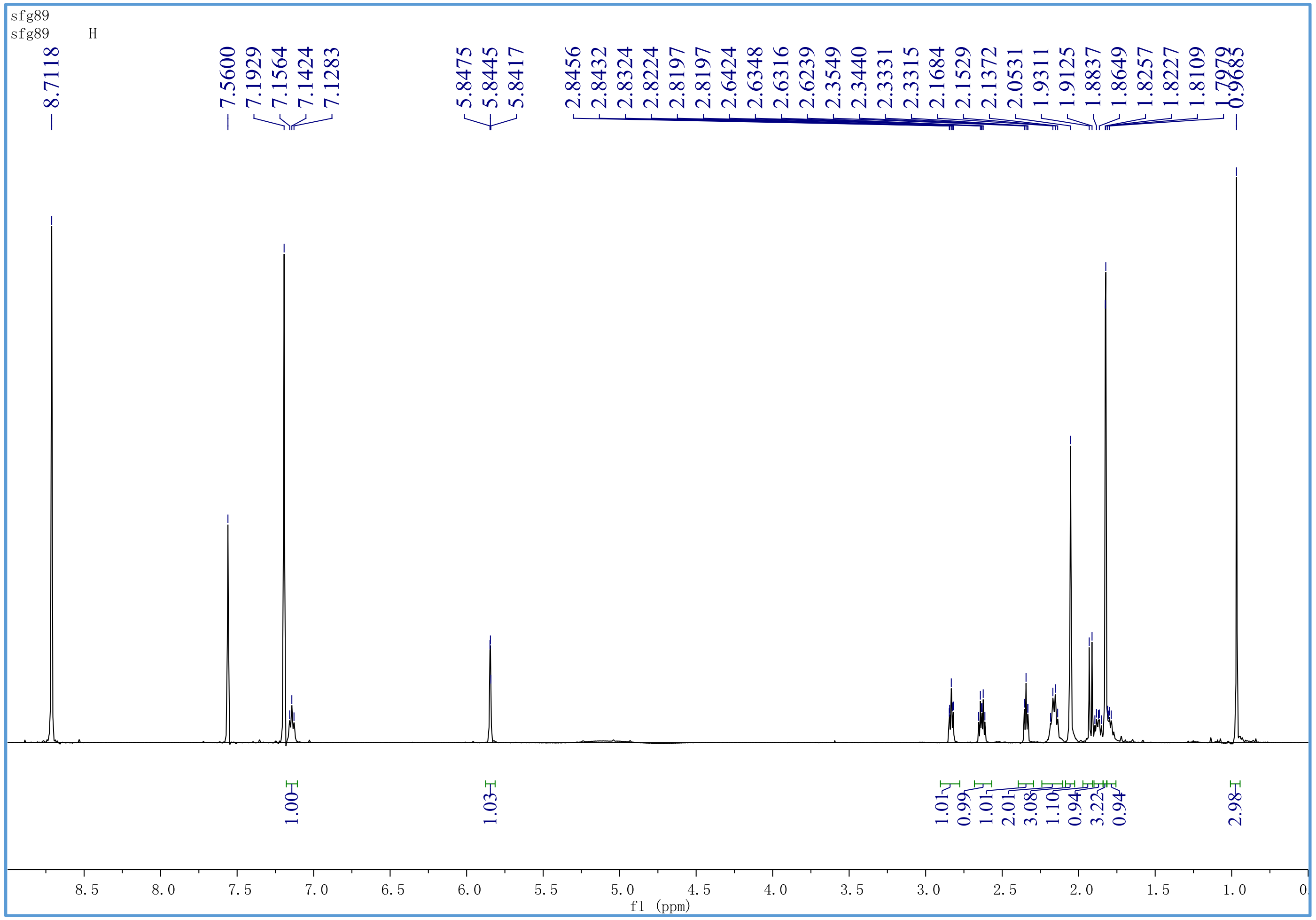

Fig. S3 ^{13}C NMR spectrum (C_5D_5N, 125MHz) of Schisanpropinoic acid (**1**)

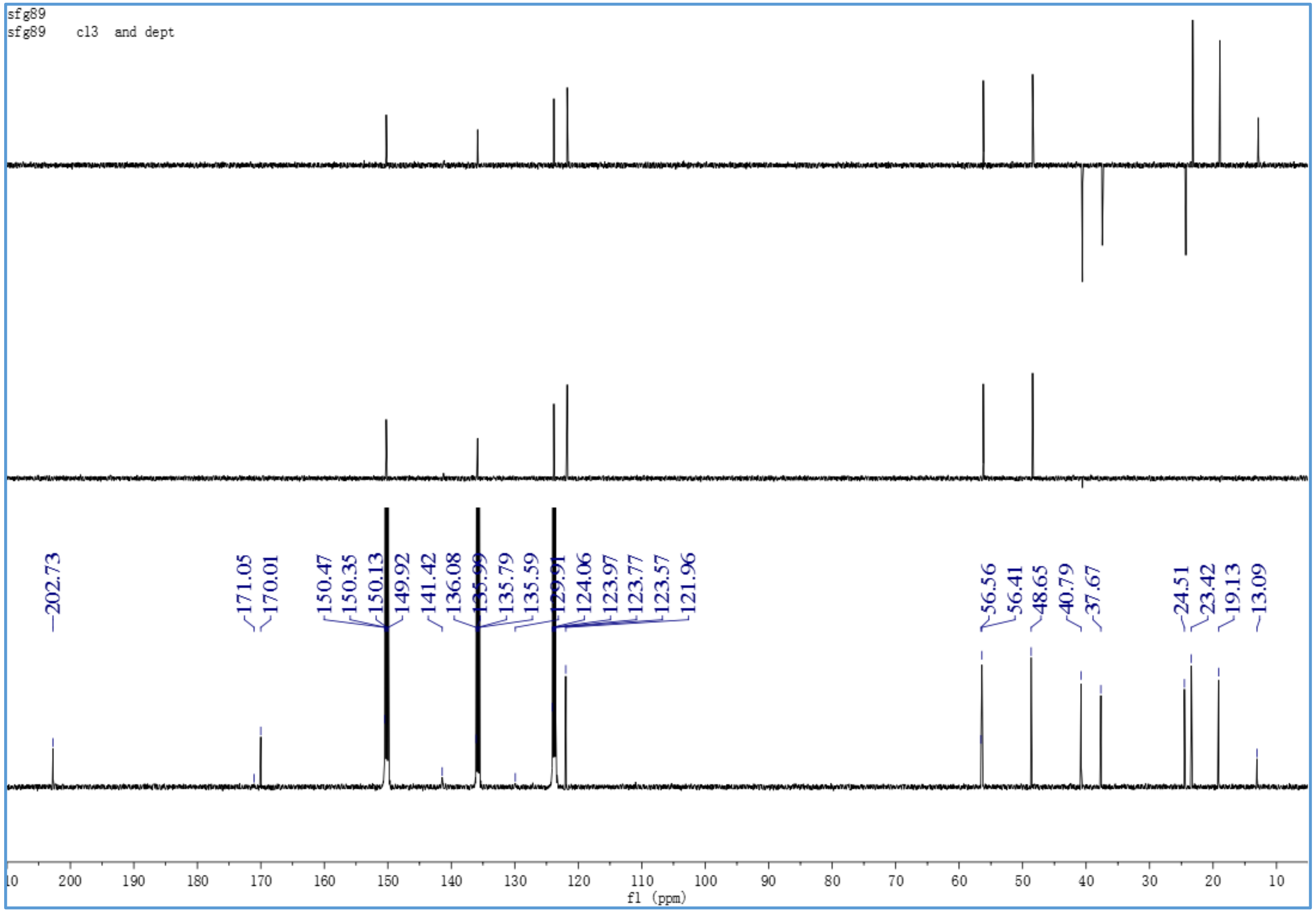

Fig. S4 HSQC spectrum of Schisanpropinoic acid (**1**)

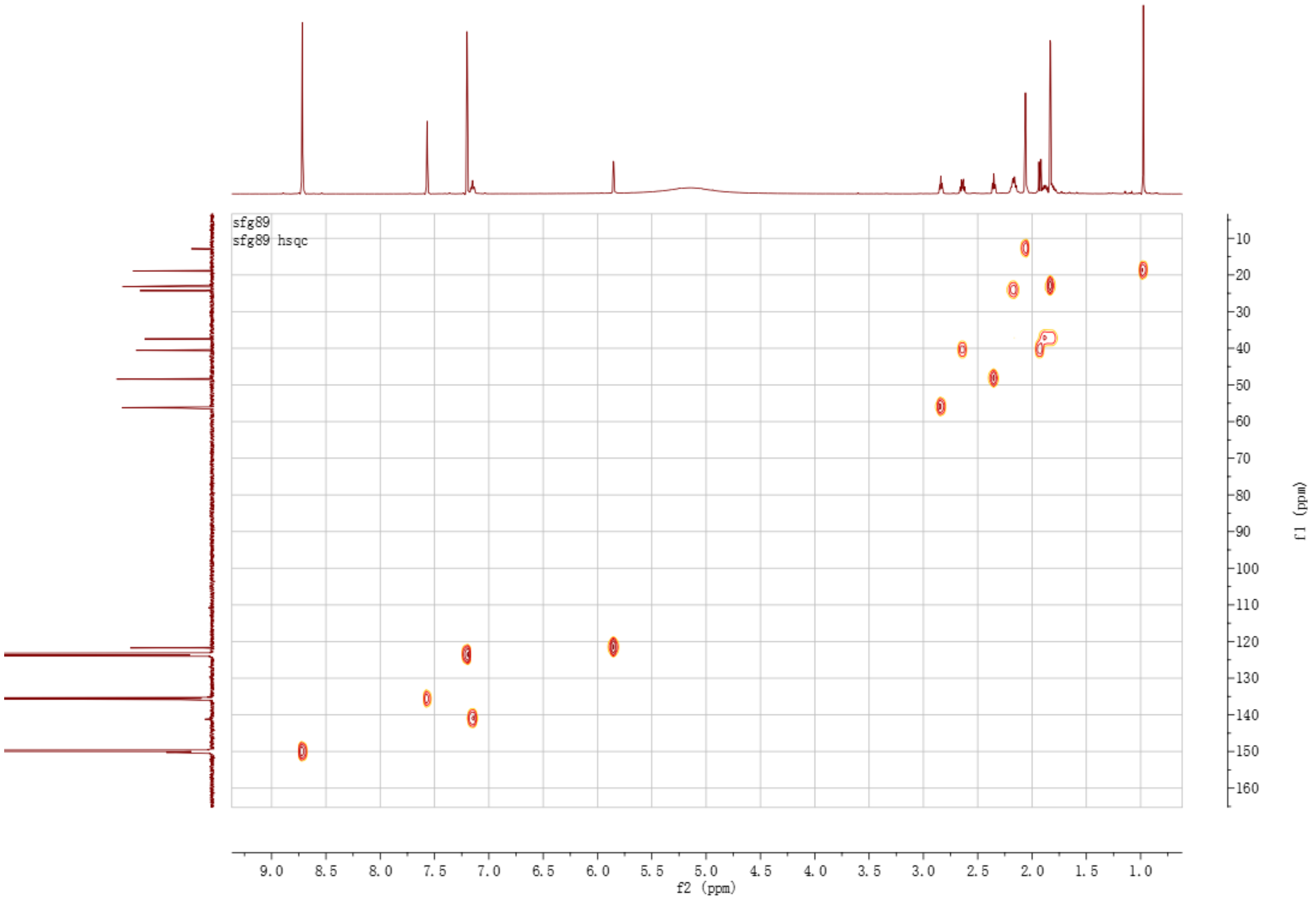

Fig. S5 ^{1}H-^{1}H COSY spectrum of Schisanpropinoic acid (**1**)

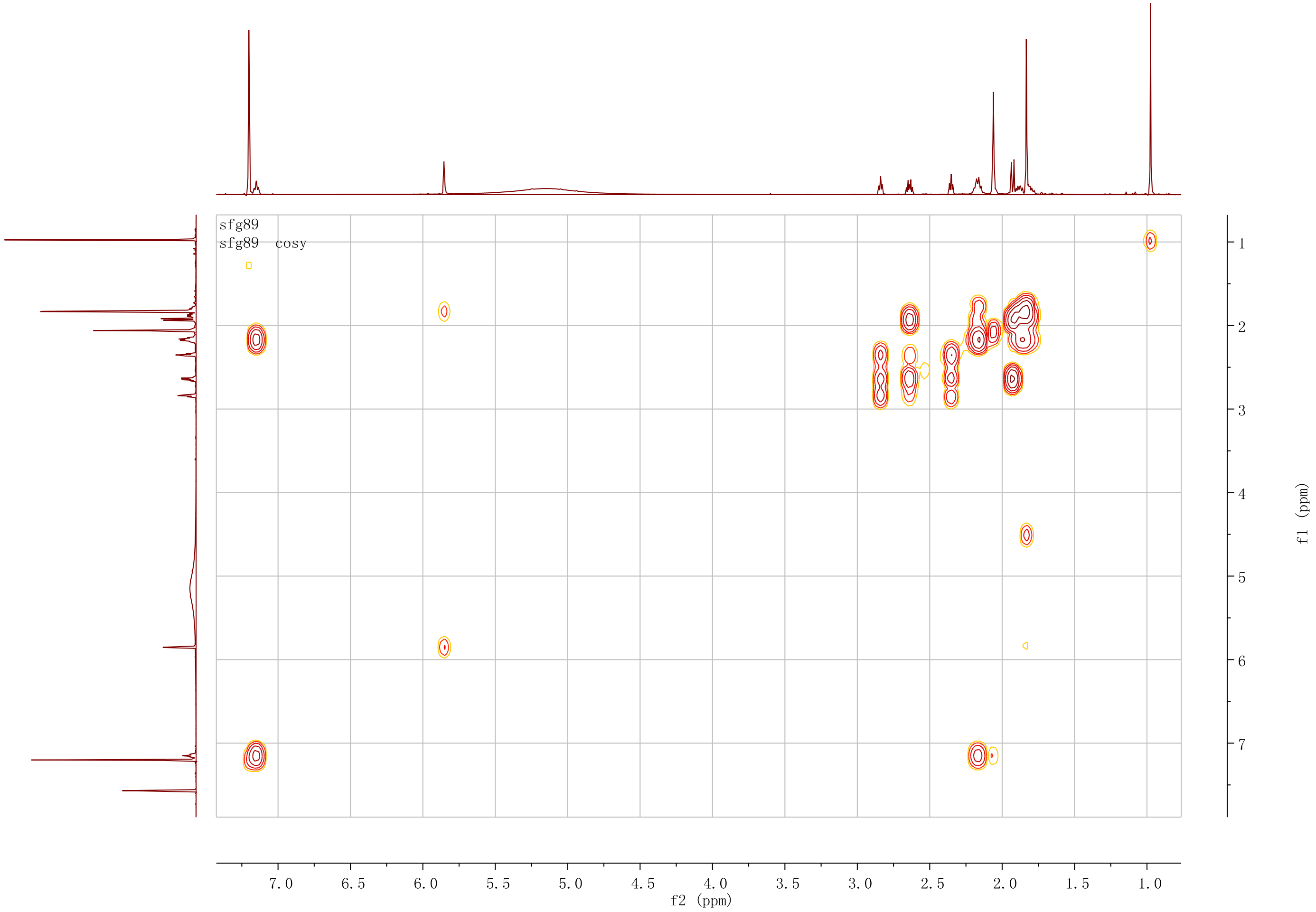

Fig. S6 HMBC spectrum of Schisanpropinoic acid (**1**)

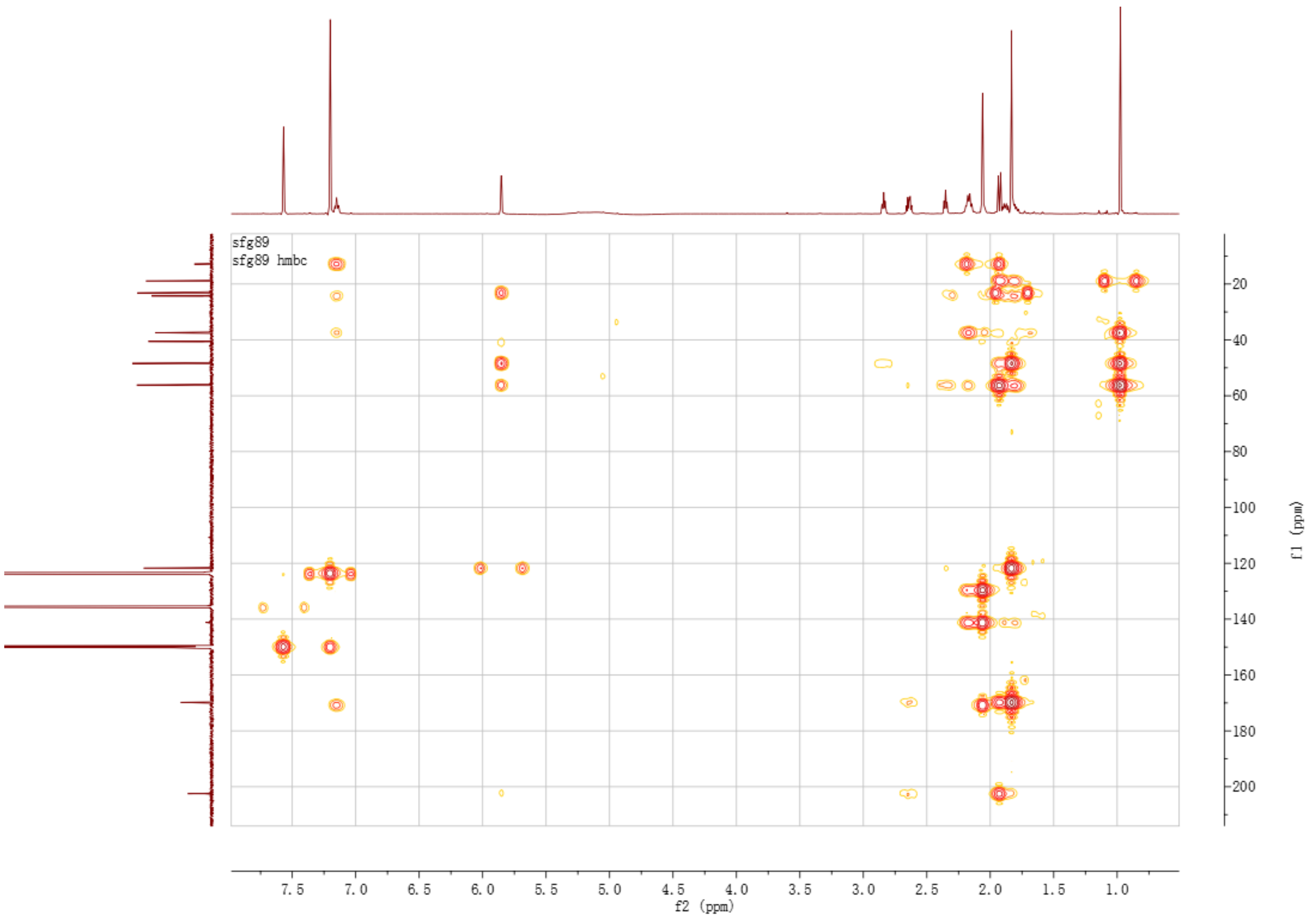

Fig. S7 ROESY spectrum of Schisanpropinoic acid (**1**)

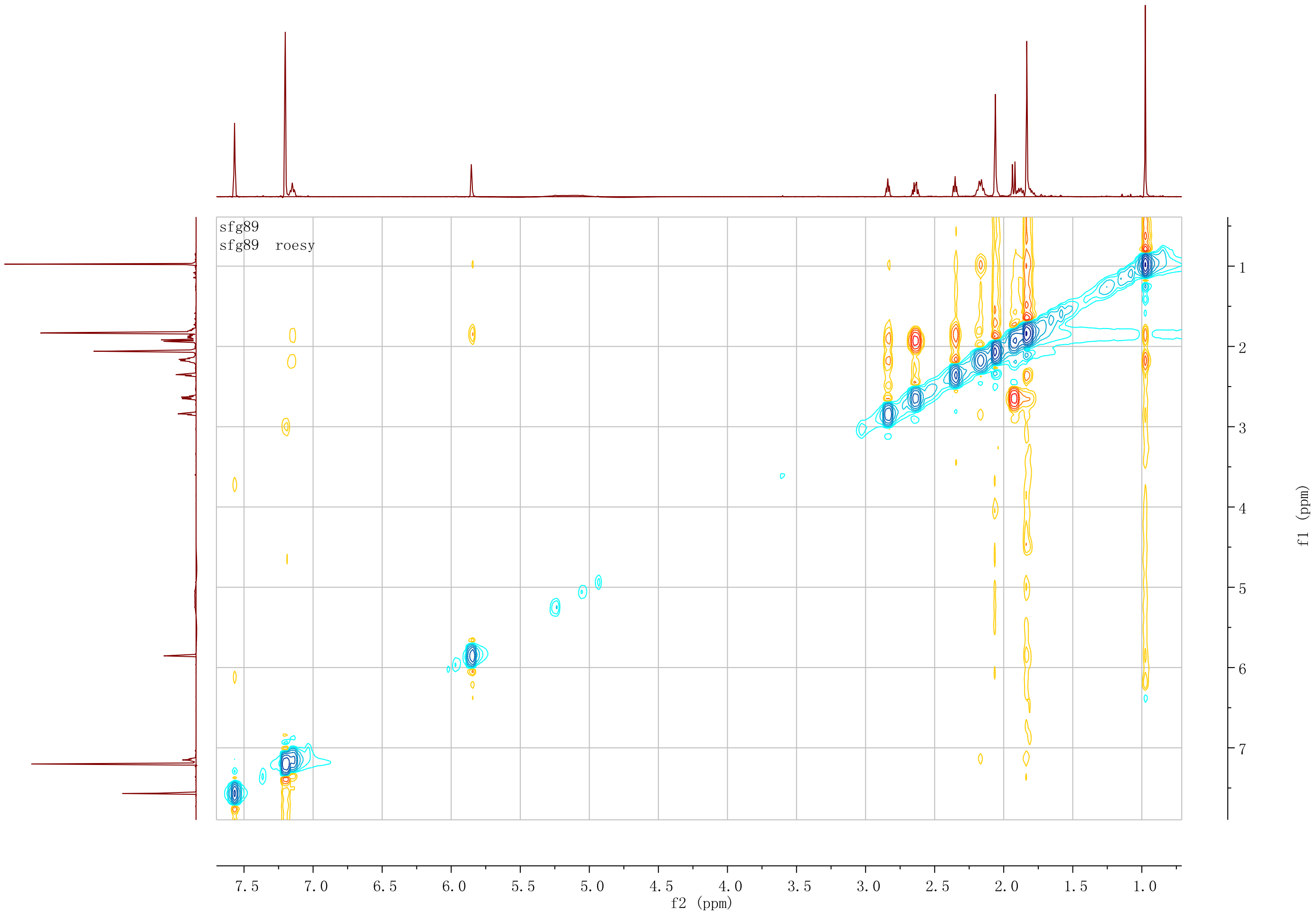

Fig. S8 UV spectrum of Schisanpropinoic acid (**1**)

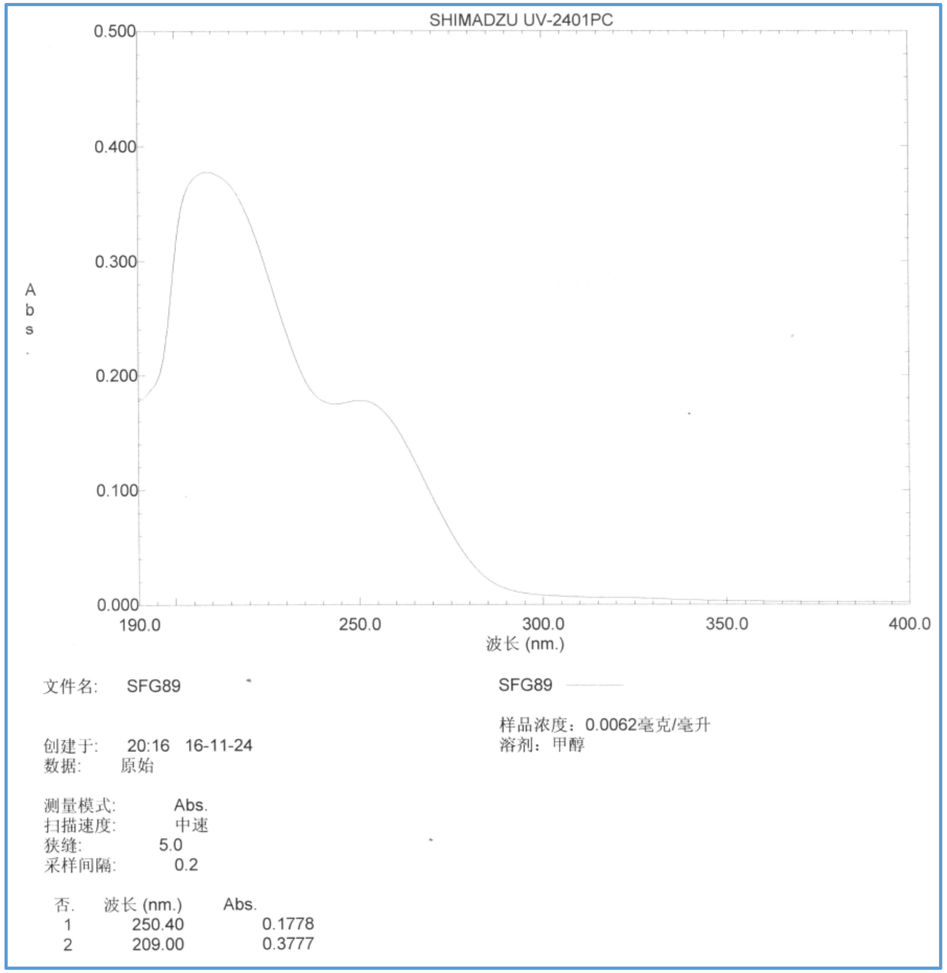

Fig. S9 IR spectrum of Schisanpropinoic acid (**1**)

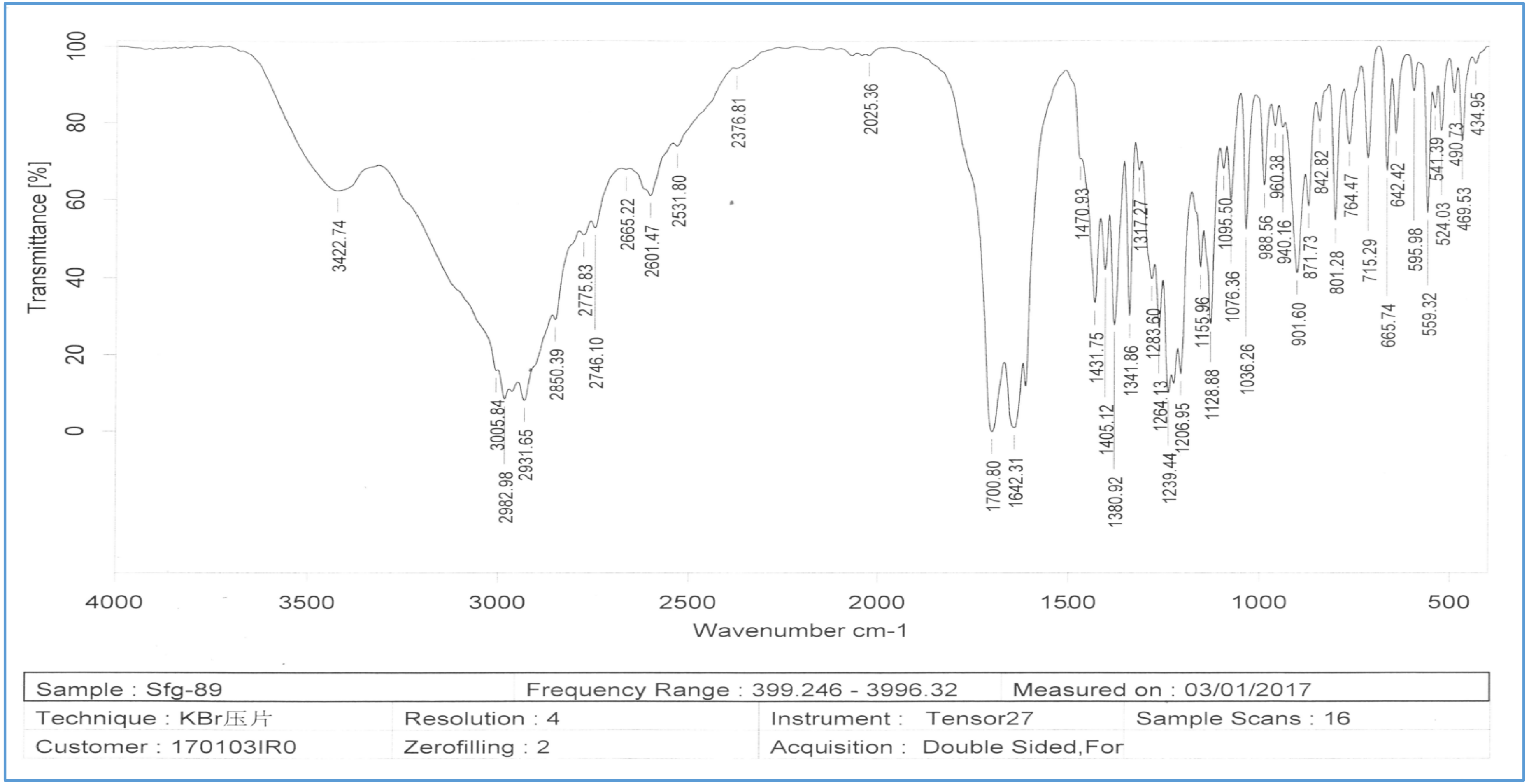

X-ray crystallographic data for **1**

Crystal data for **1**: $C_{15}H_{20}O_3$, M = 248.31, monoclinic, a = 7.1516(7) Å, b = 23.527(2) Å, c = 7.8731(7) Å, α = 90.00 °, β = 91.136(6) °, γ = 90.00 °, V = 1324.5(2) Å^3, T = 100(2) K, space group $P21$, Z = 4, μ(CuKα) = 0.687 mm^{-1}, 6450 reflections measured, 3642 independent reflections (R_{int} = 0.0501). The final R_1 values were 0.0653 ($I > 2\sigma(I)$). The final $wR(F^2)$ values were 0.1771 ($I > 2\sigma(I)$). The final R_1 values were 0.0661 (all data). The final $wR(F^2)$ values were 0.1777 (all data). The goodness of fit on F^2 was 1.167. Flack parameter = 0.0(4). The Hooft parameter is 0.03(17) for 1371 Bijvoet pairs.

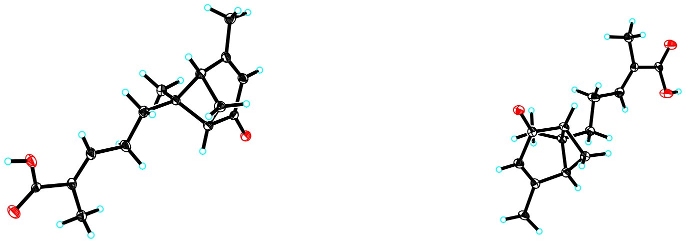

View of the molecules in an asymmetric unit.

Displacement ellipsoids are drawn at the 30% probability level.

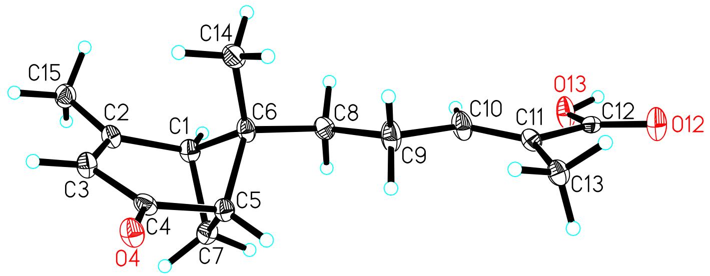

View of a molecule of **1** with the atom-labelling scheme.

Displacement ellipsoids are drawn at the 30% probability level.

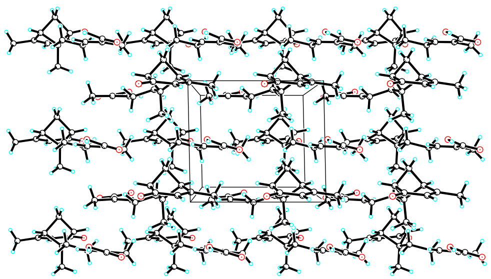

View of the hydrogen-bonded motif of **1**.

Hydrogen-bonds are shown as dashed lines.

Fig. S10 HRESIMS spectrum of schisanpropinin (**2**)

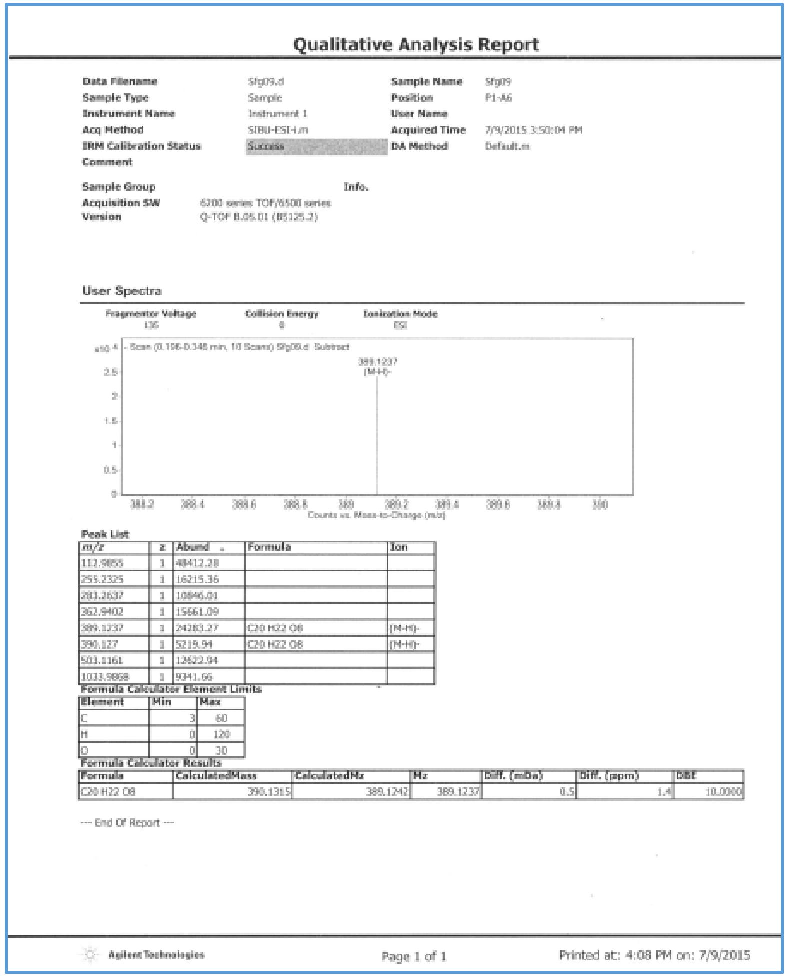

Qualitative Analysis Report

**Data Filename**	Sfg09.d	**Sample Name**	Sfg09
**Sample Type**	Sample	**Position**	P1-A6
**Instrument Name**	Instrument 1	**User Name**	
**Acq Method**	SIBU-ESI-i.m	**Acquired Time**	7/9/2015 3:50:04 PM
**IRM Calibration Status**	Success	**DA Method**	Default.m
**Comment**			

Sample Group **Info.**

Acquisition SW 6200 series TOF/6500 series

Version Q-TOF B.05.01 (B5125.2)

User Spectra

Fragmentor Voltage	Collision Energy	Ionization Mode
135	0	ESI

Peak List

m/z	z	Abund	Formula	Ion
112.9855	1	48412.28		
255.2325	1	16215.36		
283.2637	1	10846.01		
362.9402	1	15661.09		
389.1237	1	24283.27	C20 H22 O8	(M-H)-
390.127	1	5219.94	C20 H22 O8	(M-H)-
503.1161	1	12622.94		
1033.9868	1	9341.66		

Formula Calculator Element Limits

Element	Min	Max
C	3	60
H	0	120
O	0	30

Formula Calculator Results

Formula	CalculatedMass	CalculatedMz	Mz	Diff. (mDa)	Diff. (ppm)	DBE
C20 H22 O8	390.1315	389.1242	389.1237	0.5	1.4	10.0000

--- End Of Report ---

Agilent Technologies Page 1 of 1 Printed at: 4:08 PM on: 7/9/2015

Fig. S11 ^{1}H NMR spectrum (C_5D_5N, 600MHz) of schisanpropinin (**2**)

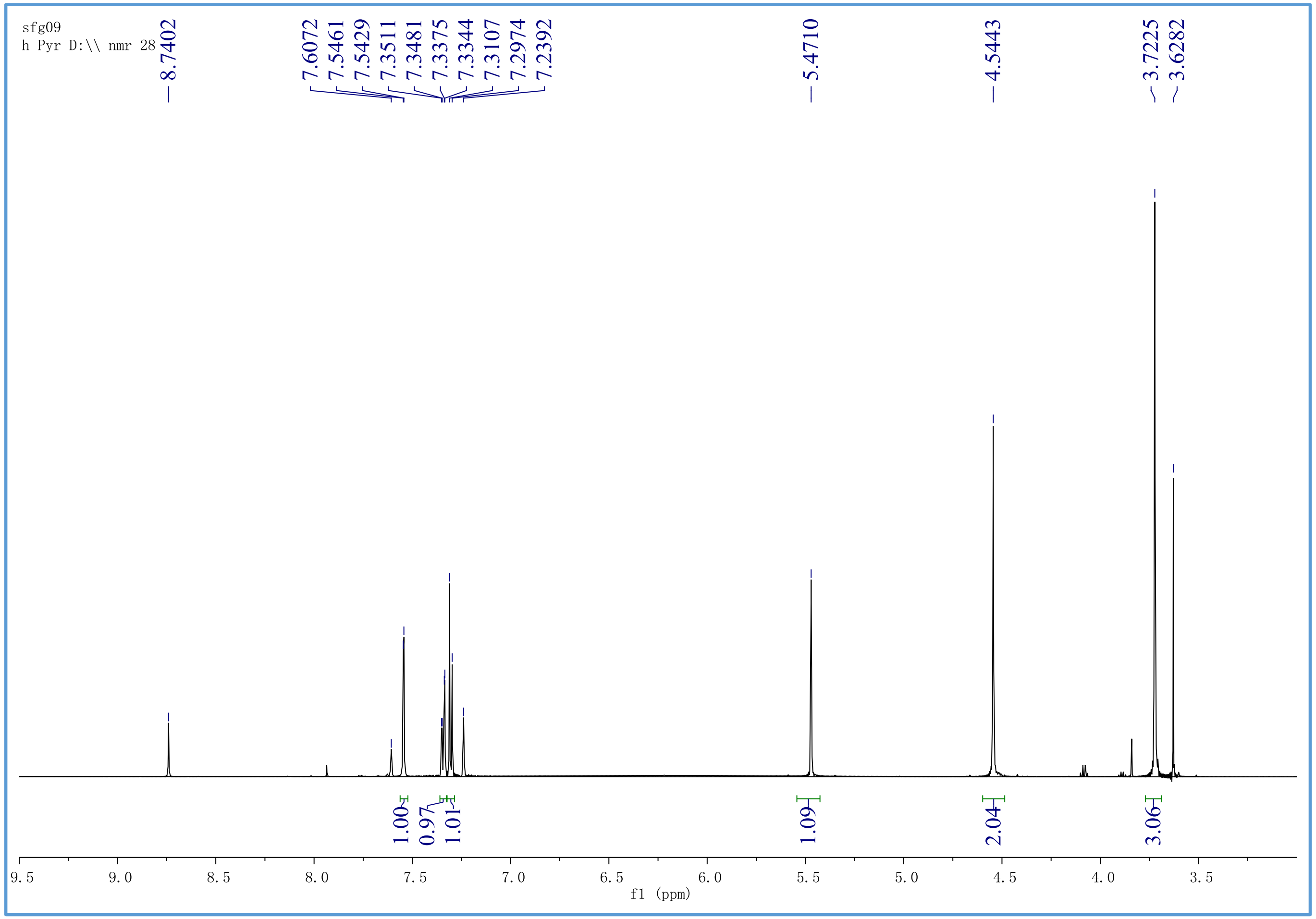

Fig. S12 ^{13}C NMR spectrum (C_5D_5N, 150MHz) of schisanpropinin (**2**)

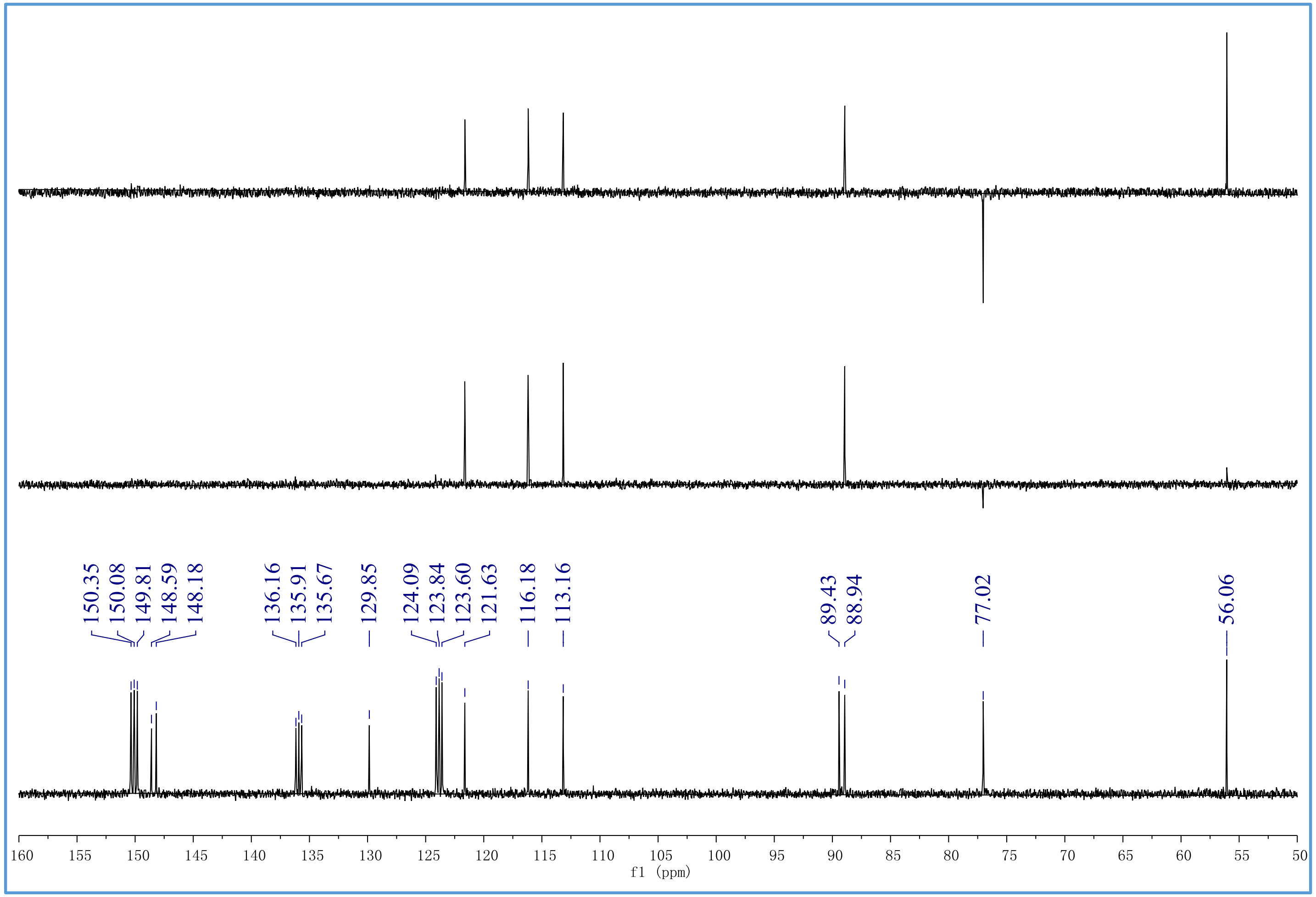

Fig. S13 HSQC spectrum of schisanpropinin (**2**)

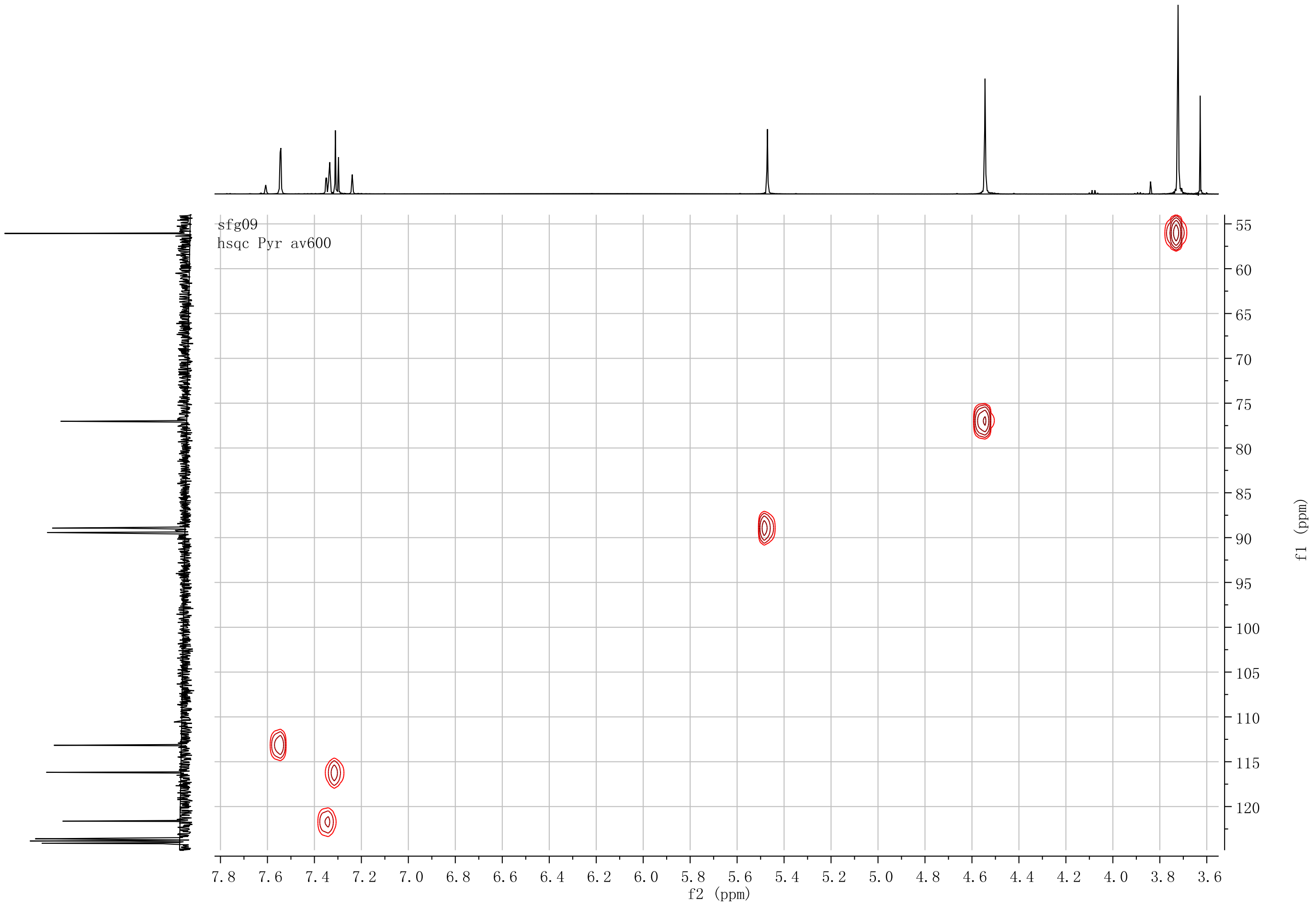

Fig. S14 ^{1}H-^{1}H COSY spectrum of schisanpropinin (**2**)

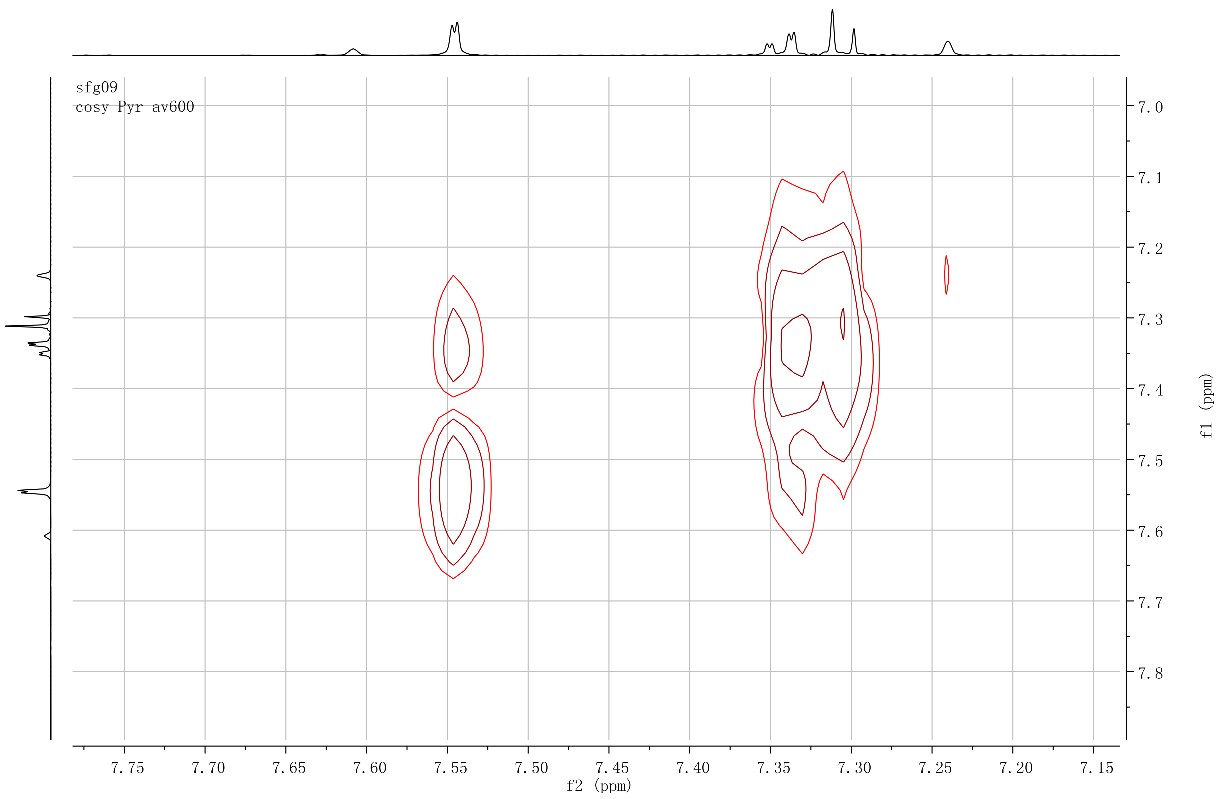

Fig. S15 HMBC spectrum of schisanpropinin (**2**)

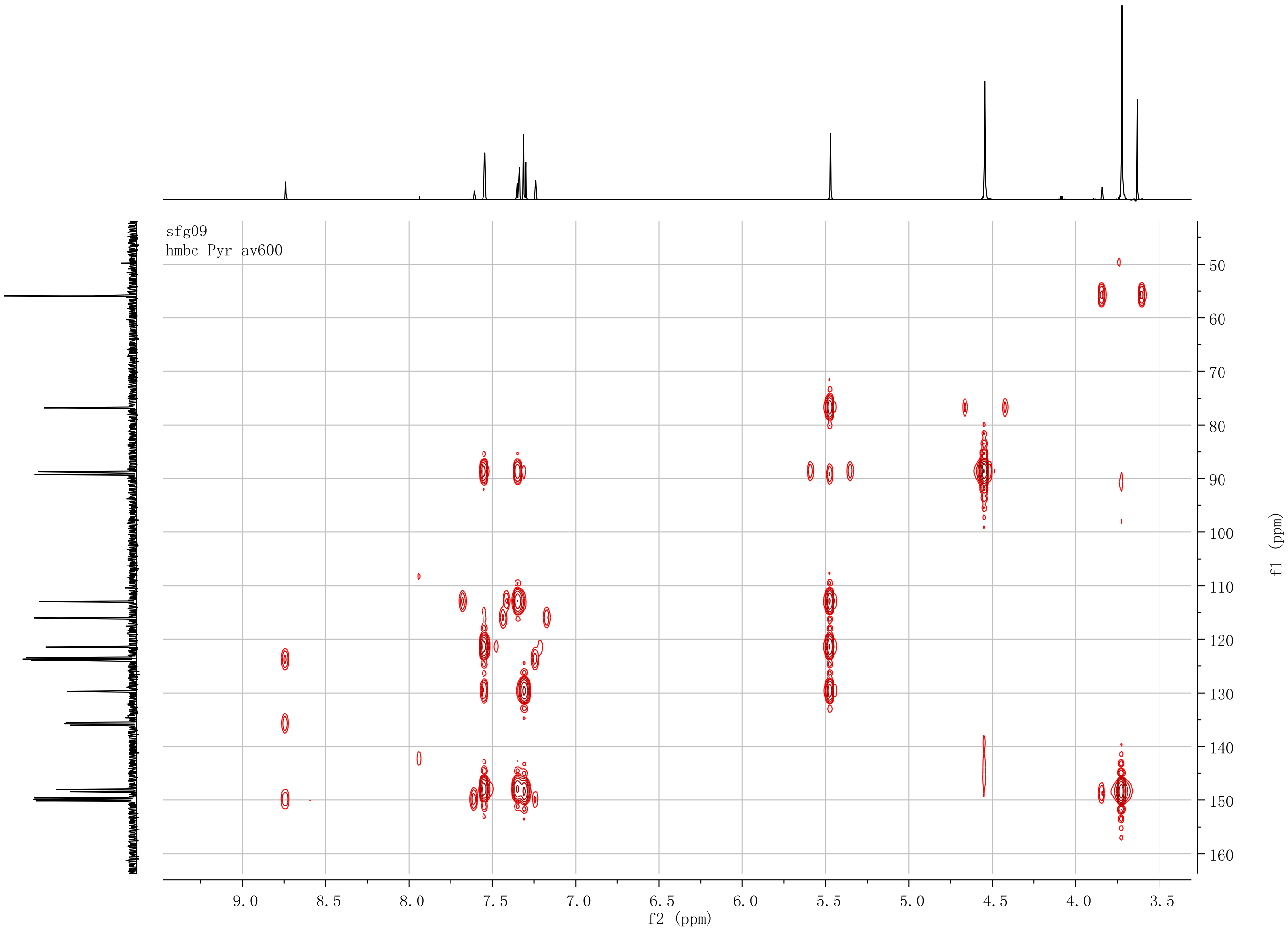

Fig. S16 ROESY spectrum of schisanpropinin (**2**)

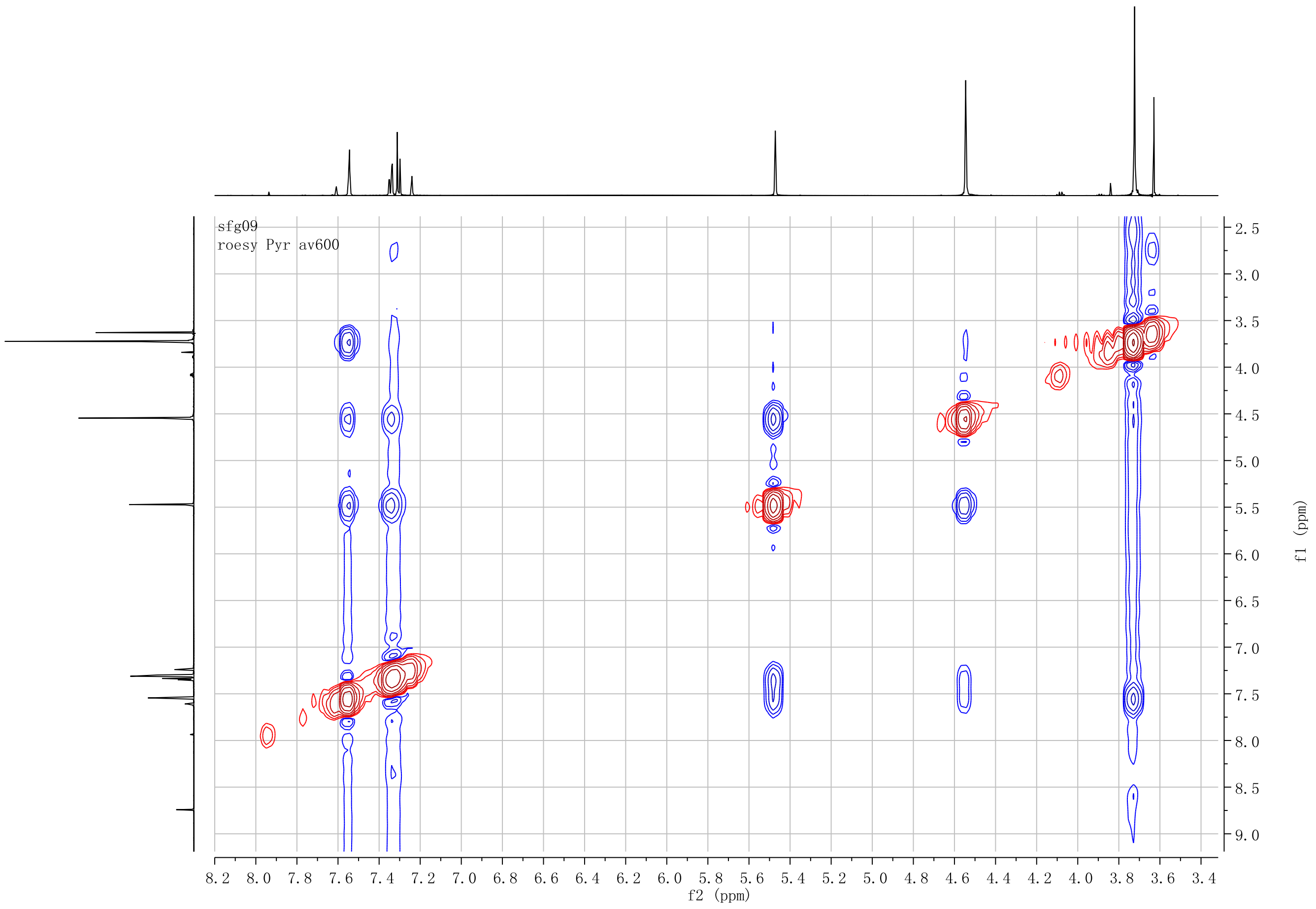

Fig. S17 UV spectrum of schisanpropinin (**2**)

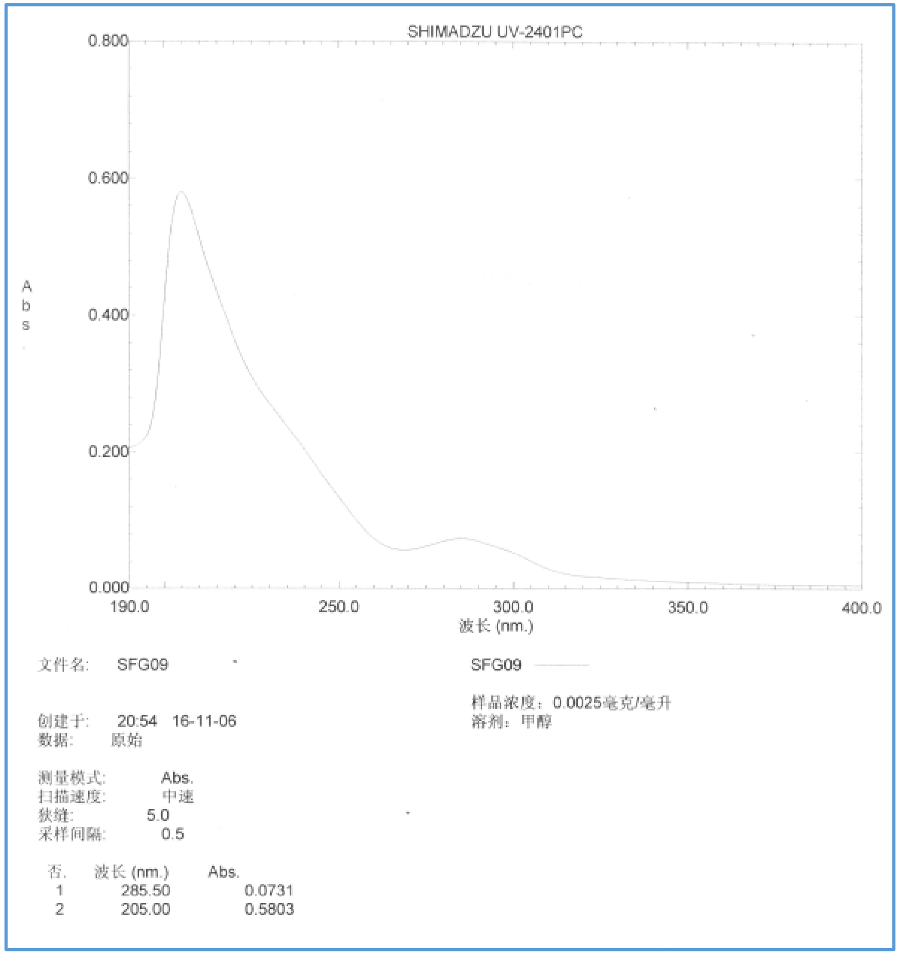

Fig. S18 IR spectrum of schisanpropinin (**2**)

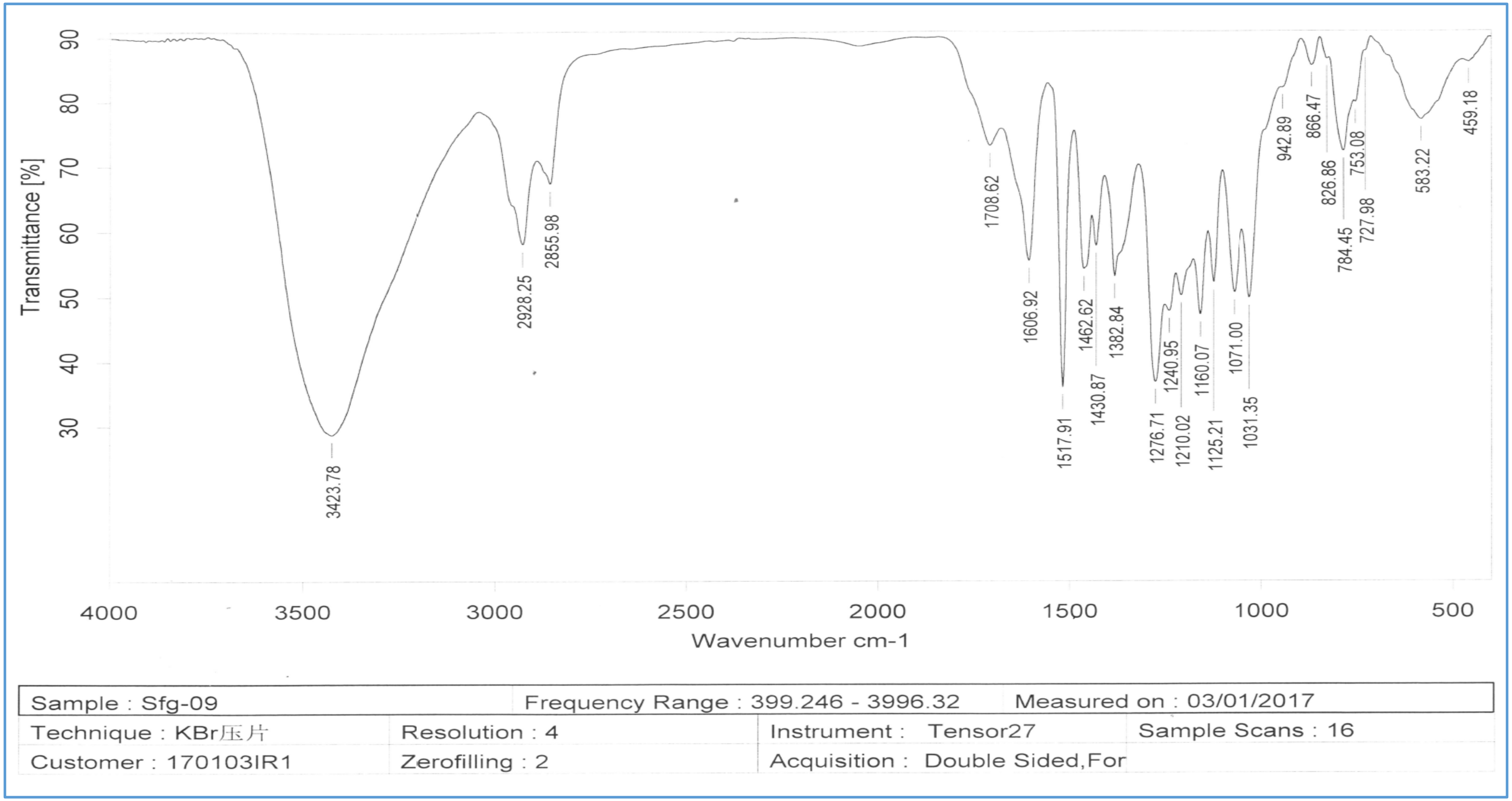

Supplement: Supplementary file 1 — (PDF 2,239 kb) [file 13659_2017_129_MOESM1_ESM.pdf]
